# Comparison of vaginal wall sling and modified vaginal wall sling for stress urinary incontinence

**DOI:** 10.1590/S1516-31802000000300003

**Published:** 2000-05-02

**Authors:** Carlos Alberto Bezerra, Marcus Vinicius Sadi

**Keywords:** Urinary incontinence, Stress, Surgery, Vagina, Incontinência Urinária, Estresse, Cirurgia, Vagina

## Abstract

**CONTEXT:**

There are several controversies about which is the best form of surgical treatment for stress urinary incontinence in women. The vaginal wall sling in its original and modified form were presented by Raz as new options for treatment of these conditions, but there is a lack of comparative clinical trials using both techniques.

**OBJECTIVE:**

To compare the effectiveness of the original and the modified vaginal wall sling.

**DESIGN:**

A comparative, prospective, non-randomized clinical trial.

**SETTING:**

Public and private health care units (Urology Division, Faculty of Medicine of the ABC Foundation, and Universidade Federal de São Paulo / Escola Paulista de Medicina).

**PARTICIPANTS:**

Twenty patients with anatomical and intrinsic sphincter deficiency stress urinary incontinence were surgically treated for evaluating the initial results of the vaginal wall sling, from February 5, 1994, to June 27, 1996.

**INTERVENTIONS:**

The patients were divided into two groups. Group A (n = 10) were treated with the original vaginal wall sling. Group B (n = 10) were treated with the modified vaginal wall sling. Both groups were statistically similar according to clinical and urodynamic parameters.

**MAIN MEASUREMENTS:**

Cure and complication rates.

**RESULTS:**

Follow-up ranged from 19 to 43 months (median = 28) for group A. The overall cure rate was 70%. Fifty per cent of the patients had urinary retention of 7 to 35 days. There were no major complications. Follow-up ranged from 14 to 26 months (median = 18) for Group B. The cure rate was 80%. Two patients had urinary retention of 7 and 55 days. There were no major complications.

**CONCLUSIONS:**

The vaginal wall sling is as effective as the modified vaginal wall sling but has a higher rate of urinary retention.

## resumo

**CONTEXTO:**

Existem diversas controvérsias sobre qual é a melhor forma de tratamento cirúrgico da incontinência urinária de esforço em mulheres. O sling de parede vaginal, em suas formas original e modificada, foi apresentado como nova opção no tratamento dessa condição, mas um estudo comparativo com ambas as técnicas ainda não foi publicado.

**OBJETIVO:**

Avaliar a eficácia dos slings de parede vaginal e modificado.

**DESENHO:**

Ensaio clínico comparativo, prospectivo, não randomizado.

**LOCAL:**

Serviços das Disciplinas de Urologia da Faculdade de Medicina do ABC e da Universidade Federal de São Paulo.

**PARTICIPANTES:**

Vinte pacientes com incontinência urinária de esforço tipos anatômica e por deficiência esfincterina intrínseca foram tratadas cirurgicamente para avaliação dos resultados iniciais do sling de parede vaginal, de 05 de fevereiro de 1994 a 27 de junho de 1996.

**INTERVENÇÃO:**

As pacientes foram divididas em dois grupos. Grupo A (n = 10) tratadas com o sling de parede vaginal original. Grupo B (n = 10) tratadas com o sling modificado. Ambos os grupos foram estatisticamente similares de acordo com parâmetros clínicos e urodinâmicos.

**VARIÁVEIS ESTUDADAS:**

Índices de cura e de complicações.

**RESULTADOS:**

O seguimento variou de 19 a 43 meses (mediana = 28) para o grupo A. O índice geral de sucesso foi 70%. 50% das pacientes tiveram retenção urinária que durou de 7 a 35 dias. Não houve complicações maiores. O seguimento variou de 14 a 26 meses (mediana = 18) para o grupo B. O índice de sucesso foi 80%. Duas pacientes tiveram retenção urinária durante 7 e 55 dias. Não houve complicações maiores.

**CONCLUSÕES:**

O sling de parede vaginal modificado tem eficácia similar ao original, que, por sua vez, tem maior índice de retenção urinária.

## INTRODUCTION

There are several controversies about which is the best form of surgical treatment for women with stress urinary incontinence.^1–5^ The surgeon's choice is based on the type and severity of incontinence, number of previous anti-incontinence procedures, hormonal status, urodynamic parameters and personal preferences.

Type III stress urinary incontinence is the involuntary loss of urine due to an intrinsically damaged urethra (intrinsic sphincter deficiency), with or without hypermobility.^1^ Patients with this type of stress urinary incontinence are best treated by slings, a procedure where a sling is harvested from fascia, muscles, vaginal wall or synthetic material and is transplanted to the suburethral area to compress and support the proximal urethra. Anatomical stress urinary incontinence is the involuntary loss of urine due to hypermobility of an intact sphincter unit. Patients with this type of incontinence may be treated with slings, but traditionally they are not, because of bladder emptying disturbances produced by this type of procedure. In this case, Burch's colposuspension is the procedure of choice.

In 1994, Young et al. modified the Raz vaginal wall sling^4^ for application in patients with anatomical and intrinsic sphincter deficiency.^6^ In this technique, there is no real sling, but two pairs of sutures placed at the level of the middle urethra and at the bladder neck.

We present a comparison of the Raz vaginal wall sling with Young's modified vaginal wall sling in patients with anatomical and intrinsic sphincter deficiency stress incontinence.

## METHODS

The procedures that follow were in accordance with the ethical standards of the committee responsible for human experimentation and with the Helsinki Declaration of 1975, as revised in 1983.

### Design

This was a comparative study of the effectiveness of two surgical techniques for stress urinary incontinence, performed prospectively and not randomized.

### Setting

Public and private health care units (Urology Division, Faculty of Medicine of the ABC Foundation and Universidade Federal de São Paulo / Escola Paulista de Medicina).

### Participants

Twenty women were surgically treated for stress urinary incontinence due to anatomical and intrinsic sphincter deficiency incontinence, between February 5, 1994, and June 27, 1996. Patients were selected according to the following criteria: a) grade 3 stress urinary incontinence (defined as severe involuntary loss of urine, which needs continuous use of pads or someother protective measure); and b) loss of urine from urethra during stress maneuver and without simultaneous detrusor contraction (defined in urodynamic study). In this way, all patients selected had the same severity of stress leak. Ten patients (group A) were submitted to the vaginal wall sling and ten (group B) were submitted to the modified vaginal wall sling. Patient selection was made without the intention of classifying types of stress uri-nary incontinence. Both types of stress incontinence (anatomical and intrinsic sphincter deficiency) were included in the study.

Preoperative investigation included complete history with physical examination and visualization of the urinary leak from the urethra during stress maneuver and urodynamics, according to the International Continence Society,^7^ and performed using a POLIMED, PL 2400 machine (Viotti Associados, São Paulo, Brazil). The urodynamic study was used to measure the intensity of incontinence, done using Valsalva leak point pressure^8,9^ and looking for detrusor overactivity that could affect the results of surgery.

Mean age, previous surgery and parity is shown in Table 1. In group A, six patients had post menopausal status, seven had periurethral fibrosis with a fixed urethra and no hypermobility of the bladder neck. In group B five patients were postmenopausal and two had a fixed urethra.

**Table 1:** Comparison of parameters: age, previous surgery and parity

Parameters	MEAN*		
	Group A	Group B	P-value
Age	53.7 (43 to 60)	51.8 (41 to 62)	> 0.05
Previous Surgery	2.9 (2 to 3)	5.7 (3 to 8)	> 0.05
Parity	1.8 (1 to 2)	1.3 (1 to 2)	> 0.05

* 95% interval of confidence in parenthesis.

The urodynamic results are seen in Tables 2 and 3. Patients 2, 4, 5, 12, 13, 17, 18 and 19 had a Valsalva leak point pressure too low to be exactly measured. But these patients leaked at a point lower than 60 cmH_2_O, which means they had intrinsic sphincter deficiency.^8,9^

### Interventions

Group A patients were submitted to the vaginal wall sling as previously described by Raz.^4^ This technique consists of incision of the anterior vaginal wall in the suburethral space, delimiting a rectangular area that is to be the sling. The sutures (polypropylene, 0) are placed in each angle of this rectangle together with periurethral tissues (urethro-pelvic ligaments) at the level of the bladder neck and with periurethral tissues and fibers of the levator ani muscle, at the middle urethra. Next, they are passed with the 0° Stamey-Pereyra needle to the supra-pubic area through the periurethral space. After suspension, the anterior vaginal wall is closed over the sling and finally, the sutures are tied.

Cystoscopy was performed routinely for checking the exact positioning of the sutures, the integrity of the urethral meatus and for the presence of sutures inside the bladder. A compressive pad was left inside the vaginal vault for 24 hours and a Foley catheter for 48 hours. For the patients with urinary retention the catheter was reintroduced for a week and then clean intermittent catheterization was instituted if normal voiding had not been resumed.

Group B patients were operated as described by Young et al.^6^ The major modification is that the vaginal wall is not harvested as a sling. Two pairs of sutures are placed through two lateral incisions at the anterior vaginal wall, excluding the epithelium. They are placed together with the periurethral tissues, at the level of the bladder neck and the periurethral tissues and levator ani muscle fibers, at the level of the middle urethra. After the suspension is done, the incisions are closed without epithelial superpositioning. The other steps of the procedure are identical.

All patients received antibiotic prophylaxis with cephalosporins before surgery, which was maintained until the urethral catheter was removed.

### Main measurements

At follow-up patients were considered cured, if completely dry; improved, if they had leakage at a lower grade (meaning leakage that did not need continuous use of pads or any other protective measure); or failed, if equal or worse. Postoperative examination included interview with one of the authors (CAB) for urinary symptoms, physical examination and trouble with sexual activity. No specific questionnaires or third party analysis were done. Patients were systematically evaluated at 1, 3, 6,12, 18 and 24 months after the procedure. Urodynamics were done only if the patient had failure or complication, and accepted this.

### Statistical methods

The Mann-Whitney test was applied for comparison of the groups, with the limit of 5% (*P* < 0.05) for the null hypothesis. The variables studied were effectiveness, defined in terms of number of patients cured or improved, and complication rates, defined in terms of voiding disturbances and sexual impairments.

## RESULTS

### Baseline comparisons

The two groups were comparable since they were statistically similar (Table 4).

### Main outcomes

For the patients in group A, follow-up ranged from 19 to 43 months (median 28); seven patients were cured or improved and 3 failed. Among the 3 failures (patients 1, 7 and 9 – Table 2), one occurred in the immediate postoperative period (patient 9), one at thirty days (patient 1) and one at 11 months (patient 7) after the procedure. Two of them (patients 1, 7) were urodynamically evaluated, confirming the persistence of stress urinary incontinence with a stable bladder. Both were reoperated and remained cured after 12 months of follow-up. The third patient (patient 9), who failed immediately, refused evaluation and treatment. She was reoperated at another institution and is still incontinent.

**Table 2:** Characteristics of group A patients

Patients	Age	Previous Surgeries	Peak flow (ml/s)	Leak point pressure (cm H_2_O)	End filling pressure (cmH_2_O)	Voiding pressure (cm H_2_O)
1	46	1 - KK	16	49	14	08
2	59	1 - KK	–	–	05	02
3	57	2 - KK-B	25	50	08	03
4	57	3 - KK-KK-B	37	–	05	02
5	50	2 - KK-B	–	–	08	02
6	57	2 - KK-G	25	90	05	02
7	33	2 - KK-R	25	90	09	12
8	53	1 - KK	–	100	08	02
9	63	2 - KK-B	35	70	12	05
10	62	2 - B-R	37	100	04	12

Legend: names of previous procedures: KK = Kelly-Kennedy; B = Burch; R = Raz; G = Gittes.

Of the patients with surgical success, five were cured and two were improved (patients 2 and 5) and demanded no further treatment. One of the latter (patient 5) had recurrent urinary tract infection for 8 months. Urodynamic evaluation revealed persistent stress urinary incontinence, a stable bladder and good emptying function, without residual volume. She has now been free from infections for 14 months.

Five patients had urinary retention after the catheter withdrawal. This condition lasted 7 to 35 days (median 14). The seven sexually active patients had no problems with intercourse after the procedure. There were no major complications except for one (patient 4), who had vaginal bleeding in the surgery, and the hemoglobin dropped from 13.4 g/dl to 9.8 g/dl. But blood transfusion was not necessary.

For the patients in group B, follow-up ranged from 14 to 26 months (median 18). Eight patients were cured (seven) or improved (one) and two patients failed. One of the procedure failures (patient 14 – Table 3) had detrusor instability, which worsened after surgery and is receiving anti-cholinergic agents. She still uses several pads a day for urge and stress incontinence. Urodynamics revealed stress urinary incontinence, normal voiding pressure, residual volume of 230 ml and reduced functional capacity due to involuntary and uninhibited contractions. The other incontinent (patient 18) had one of the sutures ruptured at surgery. Because it was felt that the other three sutures were good, it was not redone. The urodynamic evaluation revealed stress urinary incontinence with a stable bladder. She was treated by the Raz vaginal wall sling and is now continent after 11 months. Two patients had urinary retention of 7 days (patient 13) and 55 days (patient 19), respectively. Both patients resumed voiding but the second persisted with obstructive urinary symptoms, recurrent urinary infections and elevated post-voiding residual volume. After 12 months she was operated for urethrolysis and bovine pericardium sling. She is now continent but still has obstructive problems.

**Table 3:** Characteristics of group B patients

Patients	Age	Previous Surgeries	Peak flow (ml/s)	Leak point pressure (cm H_2_O)	End filling pressure (cmH_2_O)	Voiding pressure (cm H_2_O)
11	43	1 - G	25	100	02	34
12	29	1 - B	23	–	07	10
13	61	–	35	–	16	12
14	63	2 - KK-R	14	110	18	14
15	78	1 - B	–	40	22	02
16	53	–	30	100	10	05
17	57	1 - KK	15	–	12	12
18	46	2 - KK-R	–	–	12	02
19	33	1 - KK	17	–	05	35
20	55	2 - KK-B	16	90	18	15

Legend: names of previous procedures: KK = Kelly-Kennedy; B = Burch; R = Raz; G = Gittes.

**Table 4:** Statistical comparison of urodynamic parameters

Parameters	MEAN*		
	Group A	Group B	P-value
Peak flow	28.5 (21 to 36)	21.8 (15 to 28)	> 0.05
Leak point	78.4 (58 to 99)	88 (54 to 122)	> 0.05
PMCC	7.8 (5 to 10)	12.2 (8 to 17)	> 0.05
Voiding Pressure	5 (2 to 8)	14 (6 to 22)	> 0.04

* 95% interval of confidence in parenthesis. PMCC = Pressure at maximum cystometric capacity.

## DISCUSSION

Patients with stress urinary incontinence due to intrinsic sphincter deficiency may be treated by periurethral injections, artificial urinary sphincters or slings.^1,5,10–13^ After Raz described the vaginal wall sling, some authors evaluated it with promising success rates,^4,10,14^ none of which were in patients with anatomical stress urinary incontinence The original description of the procedure in 32 patients with intrinsic sphincter deficiency showed a cure rate of 88%. Four years later, the follow-up with 54 patients presented the same excellent results (91%). Two possible complications were expected with this procedure: cyst formation and vaginal shortening.^1,4^ The first is due to the superpositioning of the vaginal epithelium; the second, due to the resected vaginal wall, which could cause sexual disturbances during intercourse. Nevertheless, these two complications have not been described by any author so far, including ourselves.

The modification introduced by Young eliminates these two potential complications, since there is neither epithelium superpositioning nor vaginal shortening. But this new style of sling does have not a substantial amount of tissue positioned in the suburethral space, to compress and support the urethra. This fact may decrease the success rate of the procedure, especially in patients with intrinsic sphincter deficiency, because it is not certain that the valve mechanism, where the urethra is compressed between the sling and the pubis during stress, is maintained.^15^

Although the results were extremely favorable in Young's series, there is a lack of a single clinical trial for comparison of the vaginal wall sling with the modified vaginal wall sling in patients with both types of stress urinary incontinence.

This study proposed to compare these two procedures performed in patients with both types of stress urinary incontinence (intrinsic and anatomical sphincter deficiency). The number of patients enrolled was too short and the follow-up was done without specific questionnaires. Although we know that higher number of patients were needed and a more objective, third party, analysis should be done, some observations were possible at this initial follow-up.

In our patients, the success rate with the original Raz procedure was 70%, after a minimum follow up of 19 months (mean 28 months). With the modified procedure, an 80% success rate was obtained after 14 months (minimum) and 18 months (mean) of follow-up. Despite this lower follow-up, it is estimated that, in needle suspension procedures, and also in slings, the failures occur in the first 12 months. The initial results from slings are over 80%^5,10–14,16^ and tend to be maintained for at least a couple of years. All the failures, in group A, occurred in the first year of follow-up. Two of them were due to incorrect application of the operative technique, since when they have been reoperated, one with the same technique and the other with the fascial sling and both are now continent. The third patient was reoperated in another institution and is still incontinent, suggesting she has a severe incontinence problem that is difficult to treat with any surgical technique.

In group B one failure occurred in a patient with detrusor instability, and it is known that the results are bad when mixed incontinence is present.^8,9^ The second failure was a technical problem, since one of the sutures broke during surgery.

There were no major complications in either group, except for urinary retention, with a higher grade in group A (50% versus 20%). Despite this, only one of the twenty patients has a persistent bladder-emptying problem (patient 19, group B).

At present, the surgical treatment of stress urinary incontinence has several points of controversy. In large reviews of this theme,^19,20^ it is suggested that the best procedure for anatomical incontinence is colposuspension (Burch procedure) and for intrinsic sphincter deficiency is slings. In spite of this, we need to ask which type of sling is the best (rectus fascia, cadaveric fascia lata, vaginal wall), and whether both types of stress incontinence must be treated with slings. Some recent publications refer to slings as the best choice for all types of stress urinary incontinence.^21^ Others question this suggestion.^22^ To correctly answer these questions, more clinical trials targeting this issue are needed. This study is an initial protocol to compare two variations of vaginal wall slings, used in both types of stress urinary incontinence. Other authors have published articles with the modified vaginal wall sling, but not with a comparative trial.^3,23^ At this time, our sample and follow-up are too small to adequately answer the doubts. But it is the first comparative study with these two variations of vaginal wall sling and we are still working on it.

In patients with stress urinary incontinence, the modification suggested by Young has the advantage of eliminating the risk of cyst formation and vaginal shortening but has the disadvantage of not harvesting a substantial amount of vaginal tissue in the suburethral space. This, in our series, did not affect the initial results.

## CONCLUSION

The modified vaginal wall sling is as effective as the original vaginal wall sling, in the initial follow-up, and both have minimal morbidity. Other series of patients with greater number of cases and longer follow-up are necessary to confirm these results.
